# Supplementary material for: A Likelihood Approach for Real-Time Calibration of Stochastic Compartmental Epidemic Models
Source: PLoS Comput Biol. 2017 Jan 17;13(1):e1005257. doi: 10.1371/journal.pcbi.1005257 (PMC5240920; doi:10.1371/journal.pcbi.1005257)
Supplement: S1 File — (TAR.GZ) [file pcbi.1005257.s014.tar.gz › HSPH_Online-SI-Revision/EnKF07/n1-extreme/n1-extreme_EnKF2_table4.pdf]

1	newly 3 weeks spec	16.	9.15	{0., 17.}	100.063	1.06434	newly 3 weeks cumul	86.	67.33	{9., 91.}	98.8488	0.600217	newly 5 weeks spec	10.	3.03	{0., 6.}	94.3	1.39041	newly 5 weeks cumul	112.	75.53	{10., 111.}	95.1875	0.654291	newly 56 weeks cumul	131.	79.95	{10., 145.}	93.4885	0.693943
2	newly 3 weeks spec	15.	17.87	{1., 53.}	107.267	0.580449	newly 3 weeks cumul	66.	103.96	{16., 310.}	119.424	0.385219	newly 5 weeks spec	14.	5.98	{0., 11.}	88.5714	1.02347	newly 5 weeks cumul	87.	120.77	{16., 346.}	111.046	0.411137	newly 52 weeks cumul	117.	134.02	{18., 489.}	100.701	0.455793
3	newly 3 weeks spec	43.	17.14	{0., 17.}	98.5116	1.07719	newly 3 weeks cumul	138.	84.59	{15., 107.}	97.6304	0.67827	newly 5 weeks spec	26.	5.84	{0., 8.}	92.5385	1.39664	newly 5 weeks cumul	184.	100.64	{18., 119.}	96.6304	0.727671	newly 54 weeks cumul	247.	111.01	{21., 131.}	94.8057	0.80932
4	newly 3 weeks spec	35.	13.62	{0., 15.}	104.514	1.35228	newly 3 weeks cumul	105.	83.5	{7., 132.}	112.743	0.729552	newly 5 weeks spec	26.	3.97	{0., 5.}	99.5	1.86852	newly 5 weeks cumul	155.	94.38	{7., 174.}	105.819	0.832479	newly 53 weeks cumul	252.	99.86	{8., 178.}	96.8175	0.984871
5	newly 3 weeks spec	18.	5.45	{0., 8.}	99.7222	1.54478	newly 3 weeks cumul	78.	105.14	{7., 294.}	125.538	0.501534	newly 5 weeks spec	14.	1.11	{0., 1.}	97.0714	1.90661	newly 5 weeks cumul	116.	108.56	{7., 294.}	109.603	0.620664	newly 46 weeks cumul	161.	109.44	{7., 294.}	99.7143	0.724052
6	newly 3 weeks spec	29.	18.35	{0., 21.}	109.414	0.972487	newly 3 weeks cumul	76.	100.74	{8., 81.}	128.342	0.50224	newly 5 weeks spec	16.	6.57	{0., 10.}	103.813	1.36032	newly 5 weeks cumul	103.	118.86	{9., 97.}	124.66	0.563479	newly 51 weeks cumul	158.	128.01	{9., 165.}	110.854	0.693144
7	newly 3 weeks spec	23.	12.16	{0., 13.}	109.478	1.18733	newly 3 weeks cumul	107.	71.25	{3., 144.}	108.028	0.828886	newly 5 weeks spec	18.	5.	{0., 7.}	98.4444	1.51718	newly 5 weeks cumul	144.	83.41	{3., 144.}	104.632	0.891168	newly 55 weeks cumul	190.	93.03	{3., 144.}	101.605	0.966305
8	newly 3 weeks spec	21.	15.94	{0., 20.}	122.381	1.29859	newly 3 weeks cumul	65.	158.12	{9., 634.}	208.954	0.456061	newly 5 weeks spec	12.	5.6	{0., 11.}	106.667	1.42691	newly 5 weeks cumul	85.	173.47	{11., 686.}	182.671	0.493295	newly 49 weeks cumul	108.	182.73	{11., 704.}	159.731	0.536237
9	newly 3 weeks spec	14.	13.96	{0., 20.}	136.857	0.95416	newly 3 weeks cumul	73.	87.98	{6., 179.}	138.356	0.633572	newly 5 weeks spec	7.	4.57	{0., 4.}	130.714	1.399	newly 5 weeks cumul	94.	99.85	{6., 232.}	132.351	0.698638	newly 56 weeks cumul	112.	106.06	{7., 278.}	126.643	0.748685
10	newly 3 weeks spec	36.	6.49	{0., 12.}	93.9722	1.81767	newly 3 weeks cumul	109.	48.97	{5., 101.}	94.3945	0.895161	newly 5 weeks spec	14.	2.65	{0., 4.}	101.071	1.83562	newly 5 weeks cumul	147.	55.28	{5., 136.}	92.966	0.989289	newly 49 weeks cumul	210.	59.9	{5., 174.}	90.1429	1.11134
11	newly 3 weeks spec	14.	10.89	{0., 19.}	104.071	0.853112	newly 3 weeks cumul	74.	64.29	{11., 96.}	95.1757	0.513391	newly 5 weeks spec	6.	4.31	{0., 7.}	112.5	1.12691	newly 5 weeks cumul	95.	75.42	{13., 122.}	94.7579	0.559135	newly 55 weeks cumul	108.	87.21	{14., 184.}	98.8796	0.59058
12	newly 3 weeks spec	26.	10.1	{0., 14.}	98.7692	1.21799	newly 3 weeks cumul	91.	57.13	{9., 67.}	90.1209	0.605468	newly 5 weeks spec	9.	3.3	{0., 6.}	98.2222	1.3502	newly 5 weeks cumul	114.	65.97	{9., 75.}	92.3246	0.665262	newly 56 weeks cumul	127.	71.6	{9., 89.}	92.2047	0.68951
13	newly 3 weeks spec	35.	12.72	{0., 42.}	91.2571	1.51069	newly 3 weeks cumul	94.	124.54	{7., 424.}	139.511	0.64654	newly 5 weeks spec	33.	4.34	{0., 15.}	90.0606	1.89079	newly 5 weeks cumul	163.	136.	{8., 450.}	106.785	0.759748	newly 49 weeks cumul	326.	147.68	{8., 451.}	84.5276	0.930145
14	newly 3 weeks spec	7.	28.58	{1., 58.}	348.857	0.577309	newly 3 weeks cumul	41.	118.01	{15., 190.}	215.927	0.413544	newly 5 weeks spec	2.	15.24	{0., 56.}	723.	0.813839	newly 5 weeks cumul	46.	152.85	{18., 289.}	259.804	0.437757	newly 53 weeks cumul	53.	198.73	{19., 670.}	302.396	0.463575
15	newly 3 weeks spec	12.	5.66	{0., 11.}	109.833	1.56294	newly 3 weeks cumul	65.	51.75	{2., 137.}	107.277	0.772292	newly 5 weeks spec	12.	0.58	{0., 2.}	95.1667	1.81501	newly 5 weeks cumul	89.	54.23	{3., 148.}	98.9101	0.857539	newly 48 weeks cumul	106.	55.11	{3., 150.}	95.0849	0.911953
16	newly 3 weeks spec	31.	7.9	{0., 14.}	95.4839	1.56031	newly 3 weeks cumul	81.	101.36	{5., 272.}	123.654	0.563217	newly 5 weeks spec	20.	3.32	{0., 3.}	102.5	1.85818	newly 5 weeks cumul	118.	109.51	{5., 295.}	107.975	0.645589	newly 55 weeks cumul	180.	115.93	{5., 295.}	97.2611	0.766605
17	newly 3 weeks spec	33.	13.8	{0., 33.}	86.1818	1.05355	newly 3 weeks cumul	111.	123.46	{14., 420.}	116.072	0.536095	newly 5 weeks spec	26.	3.56	{0., 9.}	90.9231	1.5567	newly 5 weeks cumul	160.	133.92	{14., 454.}	103.263	0.61518	newly 52 weeks cumul	266.	137.92	{14., 462.}	91.0301	0.76258
18	newly 3 weeks spec	26.	13.8	{0., 17.}	104.077	1.06855	newly 3 weeks cumul	118.	71.77	{9., 120.}	95.2288	0.670203	newly 5 weeks spec	13.	4.58	{0., 9.}	97.6923	1.39449	newly 5 weeks cumul	153.	84.02	{9., 152.}	94.6275	0.71794	newly 55 weeks cumul	200.	92.97	{12., 191.}	92.845	0.788166
19	newly 3 weeks spec	32.	20.8	{0., 31.}	93.75	0.853695	newly 3 weeks cumul	144.	141.57	{15., 332.}	98.6042	0.508951	newly 5 weeks spec	11.	6.44	{0., 14.}	94.5455	0.995617	newly 5 weeks cumul	177.	160.56	{16., 372.}	95.9661	0.535521	newly 56 weeks cumul	231.	169.88	{16., 386.}	91.7403	0.604652
20	newly 3 weeks spec	8.	17.43	{0., 47.}	205.375	0.713946	newly 3 weeks cumul	57.	129.32	{11., 420.}	193.053	0.420609	newly 5 weeks spec	5.	4.72	{0., 7.}	128.8	1.02899	newly 5 weeks cumul	69.	142.41	{12., 460.}	179.232	0.444395	newly 49 weeks cumul	94.	146.57	{12., 482.}	148.287	0.511071
21	newly 3 weeks spec	40.	11.21	{0., 19.}	93.975	1.25925	newly 3 weeks cumul	123.	80.26	{5., 75.}	103.154	0.71959	newly 5 weeks spec	31.	2.14	{0., 4.}	93.0968	1.91415	newly 5 weeks cumul	179.	87.45	{6., 104.}	96.9441	0.821814	newly 52 weeks cumul	267.	90.26	{7., 108.}	93.0262	0.958617
22	newly 3 weeks spec	22.	20.33	{0., 53.}	131.591	1.25328	newly 3 weeks cumul	81.	123.	{8., 372.}	153.185	0.572843	newly 5 weeks spec	11.	8.78	{0., 15.}	140.545	1.4779	newly 5 weeks cumul	100.	144.08	{9., 598.}	152.96	0.623485	newly 53 weeks cumul	148.	161.28	{9., 722.}	133.662	0.729904
23	newly 3 weeks spec	17.	15.91	{0., 46.}	133.	1.09985	newly 3 weeks cumul	88.	83.07	{5., 145.}	115.625	0.655161	newly 5 weeks spec	9.	7.67	{0., 24.}	137.222	1.37403	newly 5 weeks cumul	105.	100.74	{5., 264.}	120.229	0.687843	newly 55 weeks cumul	155.	126.63	{5., 372.}	117.658	0.803641
24	newly 3 weeks spec	14.	12.81	{0., 15.}	133.071	1.03476	newly 3 weeks cumul	65.	66.19	{5., 62.}	113.892	0.571578	newly 5 weeks spec	7.	4.8	{0., 5.}	128.286	1.32778	newly 5 weeks cumul	79.	79.45	{5., 85.}	118.544	0.612301	newly 56 weeks cumul	97.	91.72	{5., 101.}	120.	0.668891
25	newly 3 weeks spec	37.	14.69	{0., 36.}	95.8108	1.41596	newly 3 weeks cumul	118.	146.	{10., 491.}	127.254	0.546401	newly 5 weeks spec	14.	3.15	{0., 7.}	95.7857	1.58985	newly 5 weeks cumul	153.	155.39	{10., 590.}	117.693	0.613759	newly 53 weeks cumul	253.	158.33	{10., 591.}	99.5217	0.764036
26	newly 3 weeks spec	7.	11.64	{0., 53.}	158.	0.737887	newly 3 weeks cumul	46.	99.66	{8., 253.}	168.217	0.388548	newly 5 weeks spec	5.	3.07	{0., 7.}	105.4	1.11336	newly 5 weeks cumul	56.	109.3	{8., 326.}	155.643	0.408312	newly 55 weeks cumul	70.	113.79	{8., 362.}	136.5	0.446612
27	newly 3 weeks spec	41.	13.03	{0., 20.}	94.7561	1.48789	newly 3 weeks cumul	120.	109.88	{5., 181.}	102.783	0.626407	newly 5 weeks spec	16.	5.2	{0., 8.}	101.	1.49755	newly 5 weeks cumul	166.	123.04	{6., 204.}	96.5663	0.684603	newly 50 weeks cumul	260.	133.33	{6., 204.}	90.8192	0.811275
28	newly 3 weeks spec	13.	10.89	{0., 14.}	128.846	1.2854	newly 3 weeks cumul	77.	80.53	{3., 66.}	121.26	0.648499	newly 5 weeks spec	17.	2.02	{0., 7.}	92.4706	1.77057	newly 5 weeks cumul	109.	86.52	{3., 81.}	111.303	0.755697	newly 54 weeks cumul	162.	96.7	{3., 94.}	107.16	0.900464
29	newly 3 weeks spec	7.	7.64	{0., 8.}	148.571	1.10186	newly 3 weeks cumul	71.	53.35	{4., 59.}	105.507	0.683491	newly 5 weeks spec	8.	1.55	{0., 3.}	95.375	1.54332	newly 5 weeks cumul	91.	58.22	{4., 70.}	102.593	0.753045	newly 51 weeks cumul	100.	59.75	{4., 72.}	100.53	0.772851
30	newly 3 weeks spec	10.	19.13	{0., 18.}	211.9	0.993183	newly 3 weeks cumul	42.	110.89	{6., 237.}	232.5	0.439888	newly 5 weeks spec	6.	4.75	{0., 5.}	130.5	1.19498	newly 5 weeks cumul	53.	125.36	{6., 380.}	214.377	0.471423	newly 46 weeks cumul	78.	129.61	{6., 441.}	169.372	0.570883
31	newly 3 weeks spec	10.	11.18	{0., 21.}	134.	0.909683	newly 3 weeks cumul	87.	99.96	{9., 351.}	123.356	0.543878	newly 5 weeks spec	4.	2.34	{0., 4.}	110.5	1.14734	newly 5 weeks cumul	99.	106.85	{10., 368.}	119.566	0.565182	newly 55 weeks cumul	122.	110.11	{10., 368.}	111.697	0.624555
32	newly 3 weeks spec	6.	7.05	{0., 15.}	106.833	0.616995	newly 3 weeks cumul	62.	66.45	{9., 76.}	99.4355	0.41511	newly 5 weeks spec	6.	3.63	{0., 6.}	105.5	1.1205	newly 5 weeks cumul	78.	75.32	{11., 86.}	97.0256	0.455561	newly 55 weeks cumul	82.	84.8	{12., 98.}	100.732	0.45402
33	newly 3 weeks spec	35.	11.74	{0., 13.}	102.514	1.58393	newly 3 weeks cumul	123.	106.01	{8., 107.}	111.065	0.637138	newly 5 weeks spec	29.	6.63	{0., 8.}	103.138	1.78106	newly 5 weeks cumul	183.	121.28	{8., 107.}	106.033	0.748746	newly 51 weeks cumul	278.	131.94	{9., 125.}	100.144	0.879644
34	newly 3 weeks spec	27.	26.27	{2., 46.}	120.111	0.688683	newly 3 weeks cumul	98.	138.71	{11., 242.}	131.133	0.480889	newly 5 weeks spec	12.	8.63	{0., 15.}	108.917	0.977948	newly 5 weeks cumul	137.	162.85	{11., 282.}	121.307	0.536552	newly 54 weeks cumul	165.	175.23	{11., 282.}	116.152	0.575592
35	newly 3 weeks spec	25.	23.75	{0., 74.}	127.72	0.93038	newly 3 weeks cumul	103.	135.16	{12., 463.}	143.01	0.619088	newly 5 weeks spec	20.	7.21	{0., 22.}	95.85	1.44359	newly 5 weeks cumul	144.	155.27	{12., 609.}	129.451	0.688623	newly 55 weeks cumul	192.	166.07	{12., 668.}	115.568	0.756577
36	newly 3 weeks spec	28.	26.01	{1., 71.}	117.464	0.768026	newly 3 weeks cumul	103.	118.31	{6., 350.}	113.893	0.553174	newly 5 weeks spec	9.	11.54	{0., 34.}	148.667	0.852147	newly 5 weeks cumul	131.	147.11	{6., 509.}	116.084	0.584333	newly 52 weeks cumul	175.	171.21	{7., 668.}	113.697	0.658143
37	newly 3 weeks spec	25.	34.56	{0., 102.}	178.08	1.19252	newly 3 weeks cumul	95.	89.58	{7., 334.}	114.379	0.702739	newly 5 weeks spec	12.	14.75	{0., 64.}	175.083	1.5107	newly 5 weeks cumul	128.	129.23	{7., 426.}	126.805	0.780006	newly 47 weeks cumul	171.	154.31	{7., 856.}	122.345	0.853795
38	newly 3 weeks spec	18.	22.05	{0., 52.}	130.722	0.746025	newly 3 weeks cumul	76.	123.15	{13., 516.}	133.434	0.413926	newly 5 weeks spec	9.	7.28	{0., 15.}	112.	0.949045	newly 5 weeks cumul	96.	142.52	{16., 549.}	129.042	0.448152	newly 52 weeks cumul	127.	153.09	{19., 578.}	116.748	0.509838
39	newly 3 weeks spec	23.	9.76	{0., 22.}	85.8261	1.07277	newly 3 weeks cumul	103.	93.38	{8., 257.}	90.5825	0.524708	newly 5 weeks spec	6.	2.99	{0., 7.}	98.5	1.16813	newly 5 weeks cumul	136.	101.5	{9., 284.}	85.2794	0.579653	newly 55 weeks cumul	164.	106.19	{10., 291.}	82.5915	0.623575
40	newly 3 weeks spec	14.	21.33	{0., 71.}	185.5	1.23382	newly 3 weeks cumul	66.	134.02	{3., 489.}	196.667	0.659242	newly 5 weeks spec	15.	7.75	{0., 21.}	118.333	1.64954	newly 5 weeks cumul	96.	154.37	{3., 745.}	172.302	0.754602	newly 53 weeks cumul	143.	164.98	{3., 765.}	143.175	0.866876
41	newly 3 weeks spec	19.	4.94	{0., 8.}	104.421	1.92947	newly 3 weeks cumul	71.	51.01	{3., 113.}	105.	0.793262	newly 5 weeks spec	8.	1.73	{0., 1.}	107.375	1.74844	newly 5 weeks cumul	101.	54.77	{3., 176.}	98.1089	0.905804	newly 48 weeks cumul	154.	63.35	{3., 216.}	95.1364	1.04953
42	newly 3 weeks spec	21.	14.28	{0., 24.}	111.81	0.961896	newly 3 weeks cumul	118.	106.63	{7., 201.}	113.28	0.671876	newly 5 weeks spec	14.	6.25	{0., 8.}	109.929	1.53622	newly 5 weeks cumul	148.	122.89	{7., 244.}	109.439	0.708664	newly 56 weeks cumul	159.	136.6	{9., 348.}	111.899	0.717595
43	newly 3 weeks spec	41.	9.99	{0., 18.}	93.4878	1.57839	newly 3 weeks cumul	137.	80.46	{6., 308.}	88.1168	0.736142	newly 5 weeks spec	20.	3.39	{0., 8.}	93.75	1.67612	newly 5 weeks cumul	181.	89.84	{6., 308.}	86.1878	0.798885	newly 55 weeks cumul	229.	93.99	{6., 308.}	84.1179	0.863825
44	newly 3 weeks spec	21.	22.89	{0., 69.}	147.952	1.11938	newly 3 weeks cumul	78.	107.94	{8., 411.}	133.333	0.588222	newly 5 weeks spec	11.	7.55	{0., 18.}	127.	1.41908	newly 5 weeks cumul	103.	129.17	{8., 445.}	133.155	0.654653	newly 53 weeks cumul	147.	137.57	{8., 475.}	117.884	0.753731
45	newly 3 weeks spec	42.	10.76	{0., 16.}	93.6667	1.32082	newly 3 weeks cumul	127.	69.8	{8., 111.}	89.748	0.674605	newly 5 weeks spec	16.	4.44	{0., 5.}	102.625	1.65018	newly 5 weeks cumul	166.	80.91	{9., 126.}	90.3434	0.743132	newly 56 weeks cumul	228.	89.28	{9., 145.}	90.9211	0.852793
46	newly 3 weeks spec	28.	16.58	{0., 43.}	103.286	1.23264	newly 3 weeks cumul	111.	115.25	{5., 294.}	118.73	0.640697	newly 5 weeks spec	15.	3.99	{0., 6.}	97.5333	1.54192	newly 5 weeks cumul	142.	126.82	{6., 315.}	112.155	0.697476	newly 53 weeks cumul	196.	132.37	{6., 321.}	101.291	0.783136
47	newly 3 weeks spec	37.	9.	{0., 17.}	93.7838	1.58032	newly 3 weeks cumul	141.	154.02	{4., 648.}	144.525	0.835168	newly 5 weeks spec	23.	1.1	{0., 3.}	95.3043	1.93685	newly 5 weeks cumul	188.	158.58	{4., 652.}	127.404	0.905884	newly 54 weeks cumul	283.	159.65	{4., 652.}	109.247	1.02261
48	newly 3 weeks spec	34.	10.93	{0., 15.}	101.029	1.65061	newly 3 weeks cumul	113.	109.2	{4., 202.}	127.027	0.76979	newly 5 weeks spec	18.	2.34	{0., 5.}	95.6667	1.84261	newly 5 weeks cumul	163.	116.29	{4., 246.}	111.969	0.868177	newly 54 weeks cumul	230.	120.91	{4., 252.}	102.083	0.972802
49	newly 3 weeks spec	30.	11.66	{0., 14.}	104.2	1.49351	newly 3 weeks cumul	104.	78.49	{6., 113.}	88.6635	0.573199	newly 5 weeks spec	16.	2.96	{0., 7.}	93.125	1.62547	newly 5 weeks cumul	140.	87.64	{8., 113.}	88.5857	0.647606	newly 56 weeks cumul	177.	91.81	{11., 113.}	87.4633	0.718078
50	newly 3 weeks spec	29.	18.28	{0., 16.}	113.379	1.05925	newly 3 weeks cumul	85.	105.59	{15., 150.}	93.2118	0.364569	newly 5 weeks spec	17.	7.94	{0., 10.}	116.824	1.58662	newly 5 weeks cumul	130.	125.24	{15., 156.}	90.4154	0.439689	newly 54 weeks cumul	176.	146.27	{19., 156.}	92.017	0.495132
